# Mean-Subtraction Method for De-Shadowing of Tail Artifacts in Cerebral OCTA Images: A Proof of Concept

**DOI:** 10.3390/ma13092024

**Published:** 2020-04-26

**Authors:** Woo June Choi, Bjorn Paulson, Sungwook Yu, Ruikang K. Wang, Jun Ki Kim

**Affiliations:** 1 School of Electrical and Electronics Engineering, Chung-Ang University, Seoul 06974, Korea; cecc78@cau.ac.kr (W.J.C.); sungwook@cau.ac.kr (S.Y.); 2 Biomedical Engineering Research Center, Asan Institute for Life Science, Asan Medical Center, Seoul 05505, Korea; bjorn.paulson+mtrls@gmail.com; 3 Department of Bioengineering, University of Washington, Seattle, WA 98195, USA; wangrk@uw.edu; 4 Department of Convergence Medicine, University of Ulsan College of Medicine, Seoul 05505, Korea

**Keywords:** optical coherence tomography, OCT angiography, tail artifact, mean-subtraction

## Abstract

When imaging brain vasculature with optical coherence tomography angiography (OCTA), volumetric analysis of cortical vascular networks in OCTA datasets is frequently challenging due to the presence of artifacts, which appear as multiple-scattering tails beneath superficial large vessels in OCTA images. These tails shadow underlying small vessels, making the assessment of vascular morphology in the deep cortex difficult. In this work, we introduce an image processing technique based on mean subtraction of the depth profile that can effectively reduce these tails to better reveal small hidden vessels compared to the current tail removal approach. With the improved vascular image quality, we demonstrate that this simple method can provide better visualization of three-dimensional vascular network topology for quantitative cerebrovascular studies.

## 1. Introduction

Optical coherence tomography (OCT) is a high-resolution imaging modality used to visualize the microstructure of living tissues, such as the human retina [1], and was developed due to its potential for the functional imaging of tissues [2]. Recent advances in OCT have expanded the functionality of this technique to the imaging of microcirculation in tissue beds. OCT angiography (OCTA) is an emerging technology for imaging blood perfusion in functional blood vessels, including capillaries, without the use of dyes [3]. As blood cells (primarily red blood cells [RBCs]) flow through the vessel lumen, their random motion leads to fluctuations in the light signals backscattered from the tissue, which produces temporal changes in OCT signals measured at the same spatial location [3]. The scattering dynamics can be quantified for an ensemble of OCT signals to visualize the perfused vasculature using a variety of OCTA techniques based on signal amplitude/intensity [4,5,6] and/or phase [7] as well as complex signal information [8]. OCTA is primarily used in ophthalmology and has been validated for the microvascular imaging of ocular tissues and commercialized for the diagnosis of retinal pathologies such as diabetic retinopathy [9] and age-related macular degeneration [10]. Recently, interest has arisen in capitalizing on OCTA in dermatology to image human cutaneous vasculature for the diagnosis of skin diseases such as basal cell carcinoma [11,12]. In neurology, there is interest in imaging cerebral vasculature to assess impairments in cerebral circulation resulting from stroke [13], traumatic brain injury (TBI) [14], and brain tumors [15]. 

**Figure 1:**
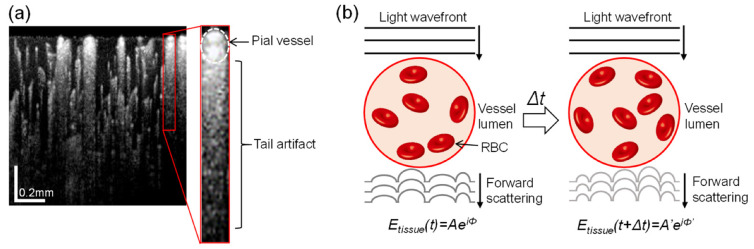
(**a**) An example of a log-scaled cross-sectional OCT angiography (OCTA) image of a mouse cerebral cortex. Inset is an enlargement showing a vertical streak (tail) trailing behind a superficial pial vessel. (**b**) Schematic illustration of the principle behind tail artifacts in OCTA images: changes in the position and density of red blood cells (RBCs) during flow over time causes variance in the forward-scattered wavefront of the light field arriving at the stationary tissue, resulting in spurious graphic artifacts behind the blood vessel during OCTA processing.

Although OCTA offers three-dimensional (3-D) vascular information, morphologic analysis of vessels is often limited to two-dimensional (2-D) projections of the volumetric vasculature because of tail artifacts. These imaging artifacts are observed as dynamic multiple scattering that appears beneath the blood vessels in cross-sectional OCTA images [16]. Figure 1a presents an example of tail artifacts in an OCTA image of a mouse cerebral cortex, in which vertical streaks (tails) trail below large superficial pial vessels (dotted oval in inset). The development of tail artifacts in OCTA images is illustrated in Figure 1b. As the light field propagates to the vessel lumen, through which RBCs flow in random orientations, the light wavefront crossing the vessel becomes distorted by the dispersion of light through the RBCs, which possess a high refractive index (~1.42), and contributes to backscattering as the light moves to the underlying tissue. After Δ*t*, however, the light passing through the vessel also undergoes dispersion but the shape of the wavefront distortion is altered because the position and density of RBCs change as they continue to move. Consequently, the optical path of light backscattered from the tissue changes after Δ*t*, resulting in temporal decorrelation in the OCT signal (*E_tissue_(t)*) at the same tissue location. This amplitude or phase difference as OCT signals travel through static tissue results in residues that appear as tails extending downward from the vasculature. Behind pial vessels with high hematocrit levels, in particular, these spurious projections can extend to the lower depths of the cortex, overshadowing true vessels. This shadowing by superficial large vessels remains as ghost images in deeper regions, complicating the delineation of vascular networks for different cortical layers.

In order to reduce tail artifacts, a few engineering and software methods have been reported. Leahy et al. employed OCT with a high numerical aperture objective to achieve a shallow depth of field that is much smaller than the scattering length, which essentially blocks multiple scattering, thus mitigating tail artifacts [17]. Despite effectively suppressing the artifacts, the high numerical aperture imaging geometry requires a time-consuming dynamic focusing process to reconstruct OCTA images [17]. You et al. intravenously injected an intralipid emulsion into mice, allowing for fewer shadowing effects in mouse somatosensory cortex images [18]. 

Furthermore, several image processing methods, including step-down exponential filtering [19,20] and slab-subtraction-based algorithms [21], have been proposed for the removal of tail artifacts. These algorithms, however, typically result in overall attenuation [19,20] or even elimination of all OCTA signals [21] in the de-shadowed region, leading to information loss for small vessels in deeper areas. Zhang et al. compensated for this issue by filtering out tail artifact decorrelation values that are likely smaller than those at the real flow [22]. However, the determination of a decorrelation threshold to remove tail artifacts is somewhat subjective and may vary between tissue beds with different vascular flow rates. Moreover, this approach may not work well for recovering small vessels such as capillaries, which can have decorrelation values below the given threshold. Recently, Baran et al. [23] reported effective suppression of tail artifacts by multiplying an *en face* OCT structural image with OCT angiograms. However, the multiplication of OCT slab might induce signal loss of capillaries not underneath the large vessels because of speckle textures seen on the *en face* OCT image [23].

In this paper, we introduce a practical approach based on mean subtraction for the removal of tail artifacts from cerebral OCTA images. Compared to existing methods, our thresholding method is adaptive based on discrete local tails, thus mitigating individual tails and balancing signal loss for capillaries behind very large vessels against the preservation of low intensity information in deeper areas. For brain imaging in mice, the proposed method preserves information about blood flow in the deep cerebral cortex, providing better angiographic contrast than existing methods.

## 2. Materials and Methods 

### 2.1. Animal Model

Animal protocols were approved by the animal care committee at the University of Washington (protocol number: 4262-01). After being placed under anesthesia using 1.5–2% isoflurane (0.2 L/min O2, 0.8 L/min air), three female C57/BL6 mice weighing 23–25 g (Charles River Laboratories, Wilmington, MA, USA) and immobilized in a stereotaxic frame were surgically implanted with cranial windows. A standard cranial window ~3 mm in diameter was centered ~1.5 mm posterior to the bregma and ~1.5 mm lateral to the midline. An open-skull cranial window was used to increase the optical penetration depth of the tissue, as described previously [19]. To create the window, a circular groove was drilled into the cranium and the central island of the skull was removed and replaced with a round cover-glass. Body temperature was maintained at 36.8 °C during the procedure with a homeothermic blanket (507220-F, Harvard Apparatus, Holliston, MA, USA). A single imaging session was performed immediately following the completion of surgery and the animals were euthanized afterward. 

### 2.2. System Setup

**Figure 2:**
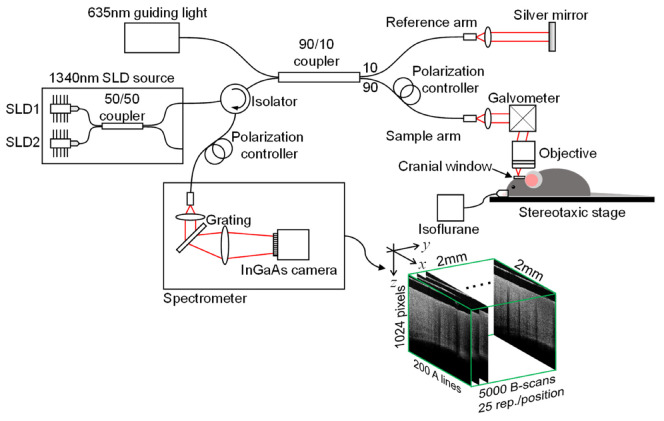
Schematic of the spectral domain OCT setup.

A 1340 nm spectral/Fourier domain OCT microscope, shown in Figure 2, was constructed for *in vivo* imaging of the mouse cerebral cortex. The light source (Thorlabs Inc., Newton, NJ, USA) consisted of two super-luminescent diodes combined by using a 10/90 fiber coupler to obtain a bandwidth of 110 nm, providing an axial (depth) resolution of 7 μm in air (~5 μm in brain tissue with a refractive index of ~1.35). 

The use of a 10× objective in the sample arm resulted in a transverse intensity profile with a full width at half maximum value of 7 μm at focus, corresponding to lateral resolution. The output light from the interferometer was routed to a home-built spectrometer with a 2048 pixel InGaAs line scan camera (Goodrich Inc., Princeton, NJ, USA) operating at 92,000 axial scans/s. The system had a measured sensitivity of 105 dB with an optical power of 3.5 mW on the sample surface.

A line trigger from the camera was used to synchronize the output of the galvanometer drive signals, ensuring that axial scans in repeated frames were acquired at the same transverse location. The operations for beam scan, data acquisition, data storage, and hand-shaking between the procedures were fully controlled using a custom software program written in the LabView language.

### 2.3. OCT Angiography (OCTA)

In order to visualize microvasculature structures through the cranial window, we used optical microangiography (OMAG), an amplitude-based OCTA method [24]. For the OMAG scanning protocol, repetitive fast scans were first performed along the x-axis (fast axis) at a scan speed of 180 frames/s, each producing 25 B-frames at the same position. One B-frame consisted of 200 A-lines covering a distance of ~2 mm. The fast scans were repeated at 200 different locations along the y-axis (slow axis), also covering a distance of ~2 mm. Completion of a raster scan within 20 s, therefore, yielded a data cube that was composed of 1024 × 200 × 5000 (z-x-y) voxels. After the OCT signal amplitudes were processed from the volumetric raw data using fast Fourier transform, an eigenvalue decomposition clutter filtering technique [25] was applied to the respective ensemble of 25 OCT B-frames to separate the dynamic scattering component representing the flow from the surrounding tissue component of the OCT signals. As a result, a data cube with only the scattering dynamic component, which is a volumetric OCTA dataset (1024 × 200 × 200 (z-x-y) voxels), was generated. The total computation time was approximately 2 minutes using a general personal computer. By applying logarithmic scaling to the 3-D OCTA dataset and collapsing it into a 2D view through maximum amplitude projection, a 2 × 2 mm^2^ *en face* image (angiogram) projecting all vessel information at specific depth ranges was finally obtained.

### 2.4. Tail Artifact Removal by Mean Subtraction

We propose the use of a simple mean-subtraction method to remove the tail artifacts in OCTA mouse cortex images. The mean-subtraction algorithm is

(1) $$OCTA_{DS}\left(i\right)=OCTA\left(i\right)-w\times \frac{1}{N}\overset{N}{\underset{k=1}{\sum }}OCTA\left(i,k\right)$$

where *OCTA_DS_(i)* and *OCTA(i)* are the *i-th* A-lines of the de-shadowed image and the original image, respectively, and *OCTA(i,k)* is a magnitude at a *k*-th pixel point of the *i*-th A-line of the original image. *N* = 1024 is the total number of pixel points of the A-line, and *w* is a constant weighting the mean value. Unlike a purely thresholding-based method [21], this algorithm depends on the total intensity in each A-line, and would thus be increasingly aggressive for A-lines with longer tails. Hence, using a subtraction method based on the mean will retain the signal strength for deeper vessels and can be combined with an additional histogram equalization step if the capillaries in deeper (poorly illuminated) regions are desired to be prominent. 

### 2.5. Comparison with an Existing Method

The step-down exponential filtering method [19,20], which is the standard for tail removal in cross-sectional OCTA images of the mouse cortex, was compared to the proposed mean-subtraction method. In the step-down exponential filtering tail removal process, the OCTA signal of the current point is attenuated by a factor proportional to the sum of the de-shadowed pixels above it as [19,20]

(2) $$OCTA_{DS}\left(i,z\right)=OCTA\left(i,z\right)\times e^{-\frac{1}{\gamma }\overset{z-1}{\underset{k=1}{\sum }}OCTA_{DS}\left(i,k\right)}$$

where *OCTA(i,z)* is an OCTA signal at an arbitrary depth position *z* of the *i-th* A-line of the original image and *γ* is a proportionality constant that controls the rate of attenuation. *OCTA_DS_(i,k)* of the exponent is a *k*-th pixel point of the *i*-th A-line, some of which were de-shadowed from the top surface to *z*−1. For comparison with the mean-substitution method, the two proportional constants *w*, *γ* in Equations (1) and (2) were manipulated to have nearly equal attenuation of tail artifacts, and the de-shadowed images were normalized without any contrast adjustment for direct comparison. 

## 3. Results and Discussion

### 3.1. Optimization of Mean-Subtraction Method Parameters for De-Shadowing of OCTA Images

**Figure 3:**
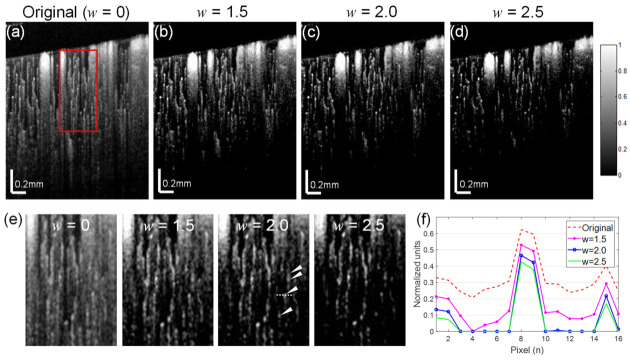
Comparison of de-shadowing performance using the proposed mean-subtraction method with a range of weight parameters w. (**a**) An original OCTA image of a mouse cerebral cortex and its de-shadowed images with *w* = 1.5 (**b**), *w* = 2.0 (**c**), *w* = 2.5 (**d**). (**e**) Enlargements of a same region (red box) in each OCTA image (**a**–**d**) showing de-shadowing effects, from which a series of capillaries (arrow heads) covered by the tail are seen for *w* = 1.5. (**f**) A graph indicating the intensity profiles taken at a section (dotted line) of the same single capillary in (**e**) showing the maximal signal-to-noise ratio near a weight of 2.0.

To understand the progression of cerebral vascular disease with OCTA imaging of the cerebral microvasculature, it is important to capture the true vessel density distribution as the vessel density varies throughout cortical layers. Here, we used single cross-sectional OCTA images of the mouse cerebral cortex obtained through a cranial window as described in the Methods section to evaluate our algorithm. The original image (Figure 3a) shows substantial tails beneath the large superficial vessels and shadow artifacts persisting into lower layers, despite the expectation that the reflected intensity decreases with depth due to light attenuation in deeper tissues. The optimal weight constant for the proposed de-shadowing method should suppress the tail artifacts while revealing the capillaries in deeper tissues. 

The performance of the algorithm with a weight constant *w* of 1.5, 2.0, and 2.5 is shown in Figure 3b–d. The tail removal performance for each of the weight conditions (1.5–2.5) is highlighted in enlarged insets indicated by a red box in the full OCTA images in Figure 3e. The deep capillary structures (see arrow heads) are resolvable in the removed tail region of the images for *w* of 2.0. Graphing a section of a single capillary (dotted line in Figure 3e) at a depth of about 550 μm shows higher intensity and contrast for all *w* settings relative to the original image (Figure 3f). The signal-to-noise ratio was maximized at about *w* = 2.0, at which the upper and lower capillaries are preserved.

### 3.2. Mean-Subtraction Eliminates Artifacts Compared to Step-Down Exponential Filtering Method

**Figure 4:**
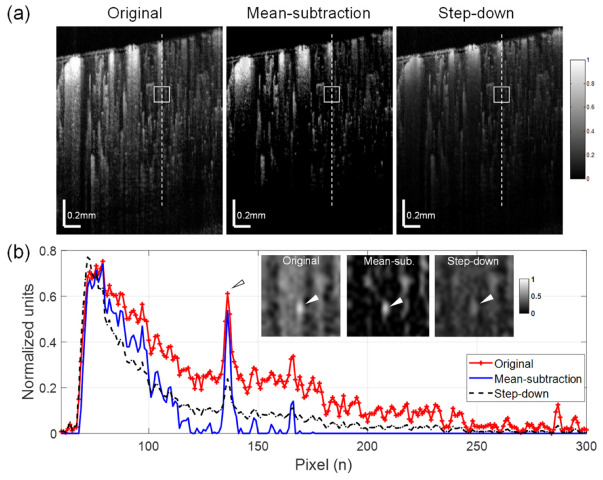
Comparisons of de-shadowing using the proposed mean-subtraction method and the well-established step-down exponential filtering method. (**a**) Normalized OCTA cross-sections of mouse cortex: original (left), processed with the mean-subtraction method (middle) and the step-down method (right). (**b**) Depth profiles taken along the dashed lines in (**a**) showing largely reduced shadow tails for both methods. However, the mean-subtraction method results in higher signal contrast for a small vessel (arrow head) in the de-shadowed region compared with the original image and the image processed with the step-down approach. Insets in the graph are enlargements of a region indicated as boxes in (**a**), where the underlying vessel (arrowhead) is brighter and more distinct with the proposed method.

In order to assess the quality of the mean-substitution method, the algorithm was compared to the step-down exponential filtering method [19,20]. The proportionality constant *γ* was empirically determined in the same manner as the optimal weighting factor *w* in our experiment. Another single cross-sectional OCTA image was selected from a different transverse scan of the mouse cortex as a test image. The results are shown in Figure 4a. Compared to the original OCTA image, it appears that the presence of shadows was significantly reduced in the OCTA images processed with both tail removal methods. This de-shadowing effect is more clearly visible in the graph in Figure 4b, which displays the depth profiles taken along the dashed line at the same location in each OCTA image (Figure 4a). Despite similar de-shadowing techniques, however, the mean-subtraction method resulted in much stronger signal intensity for small vessels (see arrow heads in Figure 4b) below de-shadowed regions compared with the step-down approach. The insets in the graph are enlarged images of a cortical region (boxes in Figure 4a) including a small vessel (arrow heads) and show the enhanced vessel contrast in the image processed with mean-subtraction. This result indicates that the proposed approach can provide better visibility for deep small vessels after the de-shadowing of OCTA images. 

### 3.3. Mean-Subtraction Method Performance on Cortical Microvasculature in En face OCTA Images

**Figure 5:**
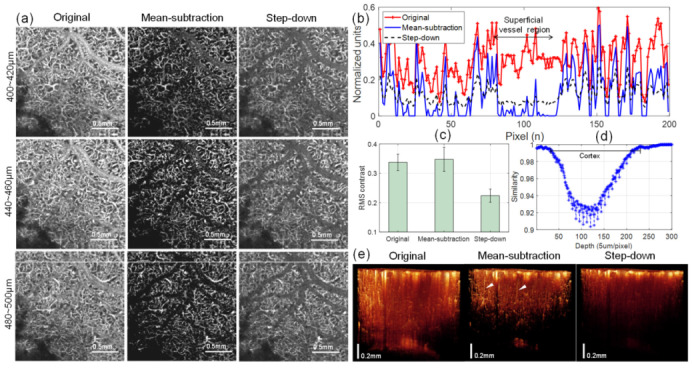
(**a**) Comparison of original and de-shadowed en face OCTA images (OCT angiograms) at different depth ranges of the mouse cortex using the proposed mean-subtraction method and the step-down method: (top) 400–420 µm, (middle) 440–460 µm, and (bottom) 480–500 µm below the cortical surface. For all de-shadowed images processed with the mean-subtraction method (second column in (**a**)), the contrast in vascularized regions is improved and the projections of overlying vessels are removed, as observed from the intensity profiles (**b**) taken along horizontal lines (white) on the angiograms at the bottom panel of (**a**). (**c**) Comparison of root-mean-square (RMS) contrasts calculated from 200 horizontal intensity profiles taken at each angiogram (440–460 μm) of (**a**). (**d**) A graph of similarity between angiograms as the cortical depth increases. Lower similarity values indicate less correlation between inner angiograms, representing the structural heterogeneity of capillary vessel networks. (**e**) 3-D reconstructions of the volumetric OCT angiograms before (left) and after de-shadowing using the mean-subtraction (middle) and step-down methods (right). The penetrating vessels (arrow heads) in the cortex are only noticeable in the image processed with mean subtraction.

*En face* images of the mouse cortex processed with the mean-subtraction method show improved visibility and shadowing effects in comparison with *en face* OCTA images (OCT angiograms) processed with the step-down method. Figure 5a compares the original, mean-subtraction-processed, and step-down-processed OCT angiograms which were generated from slabs segmented at depth ranges of 400–420 µm, 440–460 µm, and 480–500 µm from the cortical surface. Signal intensity profiles were taken along a horizontal line on the deep OCT angiograms (white in bottom panels of Figure 5a) and are shown graphically in Figure 5b. From this graph, we can see that both methods produce a significant decrease in the projection signal of the overlying superficial pial vessel at the deeper cortical region. However, the use of the step-down method also leads to a decrease in the signal strength of the regional capillaries (peaks) compared to the original image, whereas the mean-subtraction result shows the highest peaks in the graph, indicating that the small vessels at the deep cortex maintain strong signal strength in the angiogram.

To compare the effect of the processing methods on image contrast, the root-mean-square (RMS) contrasts of 200 horizontal line intensity profiles taken from each angiogram (440–460 μm) of Figure 5a were calculated. The standard deviations of the pixel intensities were provided for examining the magnitudes of vessel signals relative to the background. The results are statistically shown in Figure 5c, where the average RMS contrast with mean-subtraction (0.3476) is higher than those of original (0.3375) and step-down processed images (0.2237), indicating better vessel visibility in the de-shadowed OCT angiogram using the proposed approach. In de-shadowed images using our method, however, the cross-correlation similarity between the adjacent angiograms (Figure 5d) progressively decreases with depth, implying heterogeneity in the dense capillary vessel networks of the deeper cortex. The gap between the inner angiograms was about 5 μm, similar to the axial resolution in the tissue. However, rebound similarity was observed as the depth increased, probably due to the lack of depth contrast in our method. Furthermore, in the 3-D rendering of the volumetric OCT angiography (Figure 5e), the de-shadowed result using the mean-subtraction method depicts vascular structures in the cortex such as penetrating vessels (arrow heads) that are obscured in the original and step-down results. 

### 3.4. Limitations and Implications

The proposed algorithm is a simple and effective approach to remove tail artifacts in deeper layers of OCTA images of the mouse cortex. However, a limitation of this approach is that it may induce partial loss of capillaries close to the middle of the tail artifact in OCTA images. At points closer to the center of the vessel lumen, the tail below the vessel becomes longer, increasing the mean value of the line. The subtraction of the mean value, therefore, may cause the loss of capillaries with signals below the mean value. This effect is more significant for capillaries below larger surface vessels (>100 μm) or for deeper capillaries with weaker signal strengths. This limitation might be mitigated with the help of engineering techniques that can physically reduce the tail artifact; for instance, an alternative hardware-based method, dynamically focused optical coherence microscopy angiography with a high numerical aperture, has successfully been applied to reduce the tail artifacts in synthesized angiograms [17]. Another potential hardware-based approach is the reduction of the time interval between B-scans. According to Tang et al. [26], OCT signal decorrelation beneath vessels increases with B-scan time intervals of 6 ms or longer. Therefore, the decay of vessel tails can be minimized by reducing the time interval between inner B-frames to 1 to 4 ms [26]. However, this approach may lead to signal loss for some capillaries due to intermittent slow flow in single passages.

The brain contains a complicated vascular network that varies in density throughout the cortical layers [27]. Within each layer, the capillary vascularity can be modulated by pathological, physiological, and environmental factors, such as aging [28], hypoxia [29], and hypertension [30]. Accordingly, accurate quantification of the cerebral microvasculature in deeper layers of the brain is essential to understand the progression of neurological pathologies. In this work, we demonstrated an algorithm to suppress shadow artifacts in OCTA images acquired from the deeper layers of the mouse cerebral cortex *in vivo*. The proposed algorithm noticeably increases the contrast in cross-sectional images (Figure 4b) as well as in *en face* images (Figure 5b), resulting in improved visibility of capillaries hidden behind larger vasculature and reduced expression of shadow artifacts. Improved visualization of capillaries or small vessels with this approach can enable more accurate analysis of vessel networks in deeper cortical layers. Therefore, this algorithm may have important applications in the study of cerebrovascular reactivity using experimental brain vascular disease models.

## 4. Conclusions

In conclusion, we introduced a simple post-processing method based on mean subtraction to effectively suppress vessel tail artifacts in microvascular angiograms, preserving contrast in deeper vascular regions. Application of this method to volumetric OCT angiography images of the mouse cerebral cortex obtained through a cranial window led to the reduction of tail artifacts and enhanced the contrast for parenchymal capillary vasculature. In the future, this method may be further applied to quantitatively measure changes in vessel density or tone, which may have applications beyond the neuroimaging field, such as in ophthalmology.
